# MSP-N: Multiple selection procedure with ‘*N*’ possible growth mechanisms

**DOI:** 10.1371/journal.pone.0224383

**Published:** 2019-12-12

**Authors:** Pradumn Kumar Pandey, Mayank Singh

**Affiliations:** 1 Computer Science and Engineering, Indian Institute of Technology Roorkee, Roorkee, Uttrakhand, India; 2 Computer Science and Engineering, Indian Institute of Technology Gandhinagar, Gandhinagar, Gujarat, India; University of Warwick, UNITED KINGDOM

## Abstract

Network modeling is a challenging task due to non-trivial evolution dynamics. We introduce multiple-selection-procedure with ‘N’ possible growth mechanisms (*MSP-N*). In *MSP-N*, an incoming node chooses a single option among *N* available options to link to pre-existing nodes. Some of the potential options, in case of social networks, can be standard preferential or random attachment and node aging or fitness. In this paper, we discuss a specific case, *MSP-2*, and shows its efficacy in reconstructing several non-trivial characteristic properties of social networks, including networks with power-law degree distribution, power-law with an exponential decay (exponential cut-off), and exponential degree distributions. We evaluate the proposed evolution mechanism over two real-world networks and observe that the generated networks highly resembles the degree distribution of the real-world networks. Besides, several other network properties such as high clustering and triangle count, low spectral radius, and community structure, of the generated networks are significantly closer to the real-world networks.

## Introduction

We witness a variety of complex social systems and non-trivial interactions among actors of the network [1]. In real-world networks, actors are represented as nodes, and interactions among actors are represented as edges. The definition of nodes is contextual, for example, in World Wide Web (WWW) network [2], web pages are considered as the nodes while in protein-protein interaction network [3, 4], proteins act as nodes. This diversity results in the non-trivial distribution of fundamental properties including degree distribution [5, 6], clustering coefficient, triangle distribution [7], small-world property [8–14], low average path length, assortativity [15–17], and community structure [18]. The *degree* of a node in a network is the number of connections it has w other nodes, and the *degree distribution* is the probability distribution of these degrees over the whole network. The degree distribution varies from power-law (a.k.a., *scale-free*) [1, 19, 20] to exponential. Similarly, a *clustering coefficient* is a measure of the extent to which nodes in a graph tend to group together [21]. Clustering coefficient varies from meager value to very high-value [1]. Assortativity represents a tendency of nodes to connect to other nodes that posses similar properties, for example, the tendency of actors to connect others having similar degree. In literature, for different networks, assortativity varies from negative (disassortative mixture) to positive values (assortative mixture) [15–17]. A network is considered to have *community structure* if the nodes of the network can be easily grouped into (potentially overlapping) sets of nodes such that each set of nodes is densely connected internally. Modularity [22] is often used in optimization methods for detecting community structure in networks.

In past, several classical growth mechanisms [8, 23–33] have been proposed to explain the network properties. Some of these interesting hypotheses include scaling behavior of the degree distribution, node aging, cost of link formation, randomness and preferential attachment in link formation. Interestingly, in 1955, Herbert Simon [34] confirmed the existence of ‘*rich get richer*’ phenomena leading to power-law tail in degree distribution of several real-world networks. Later, Derek De Solla Price [35] proposed a similar idea in the context of bibliographic networks. Albert and Barabási [8] (hereafter *BA model*) proposed a degree-based preferential attachment process that beautifully explains power-law tail. The fundamental intuition behind the preferential attachment is that the probability (*p*_*ij*_) of connecting a newborn node *i* to an older (pre-existing) node *j* is an affine function of the degree of node *j* given by

$$\begin{array}{c} p_{ij}=\frac{k_{j}+x_{j}}{\sum_{l}k_{l}+x_{l}}. \end{array}$$ (1)

where *x*_*j*_ is a constant and *k*_*j*_ is the degree of node *j*. If *N* denotes the total number of nodes, then *l* ∈ [1, *N*]. The BA model proposed a constant value (= 3) for the power-law exponent with the clustering coefficient (O(1/n)) vanishing as the network grows. However, majority of the real-world networks possess a wide range of exponent values and non-zeros clustering coefficients. Some of the interesting real-world networks that follow power-law distribution are phones call graphs [36], Internet [37], Web [8, 38, 39], click-stream data [40], who-trusts-whom social network [41], etc.

Similarly, random attachment mechanisms emphasize on the uniform-random attachment of nodes and edges. Empirically, they generate networks with lower clustering coefficient along with Binomial or Poisson degree distribution [1]. In 1998, Watts and Strogatz [14] proposed a variant based on the random rewiring of a regular network. This mechanism generates networks with higher clustering along with a bell-shaped degree distribution. Additionally, several other network models have been proposed in the literature that capture different statistical and spectral properties of real-world networks [8, 42–48]. We claim that these mechanisms do not perform well in isolation with each other. Thus, we encompass above mechanisms in a more generic network growth model based on *Multiple Selection Procedure* (MSP).

In real-world networks, a node can interact with other nodes in more than one possible linking mechanism. A simple analogy is people traveling from one place to another using different modes of travel. Each person (node) may use a different mode of traveling, depending on his financial conditions, comfort level, age, popularity, and delay estimate. Similarly, MSP-N assumes the availability of N-possible linking mechanisms. An incoming node chooses one out of N mechanisms.

**Fig 1:**
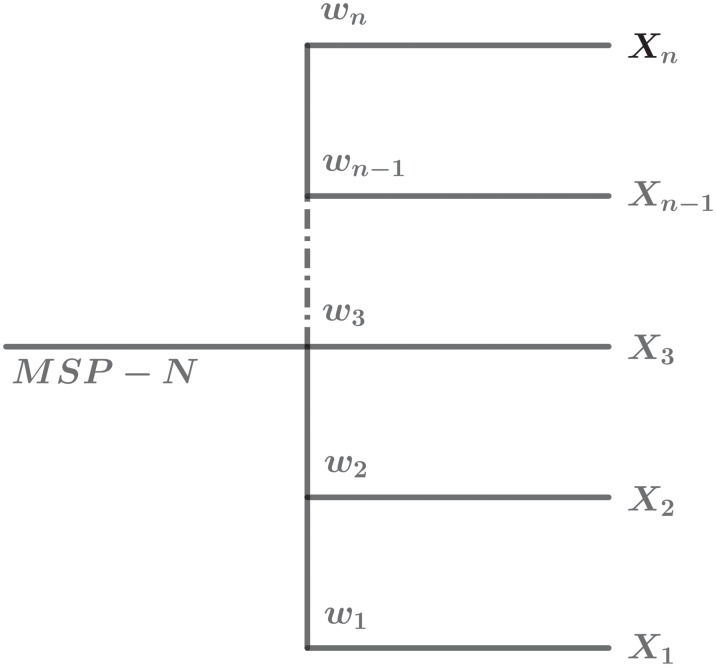
Graphical representation of Multiple-Selection-Procedure (MSP-N). Each incoming node can choose one out of N mechanisms for link formation.

Fig 1 shows the graphical representation of MSP-N. Specifically, we derive and investigate MSP-2 that encompasses preferential attachment [8], cost of linking [29, 30], local dynamics [31], and aging [32].

We show that MSP-2 leads to non-trivial characteristic features of a larger class of social networks, including networks with power-law degree distribution, power-law with an exponential decay (exponential cut-off), and exponential degree distributions. The generated class of scale-free networks (power-law degree distribution) shows power-law exponent (1 + 1/*β*) in the range (2, ∞) and phase transition in average connectivity, derived as (2*β* − 1), at *β* = 1/2. We also present bounds on the average connectivity of the networks under different settings of model parameters. We demonstrate the high similarity between generated networks against real-world networks. The simulated networks show high clustering, slow growth of hubs (high degree nodes), edge densification, and community structure—presenting a good resemblance with real-world networks. We also show that the edge densification restricts the growth of the diameter in a random network.

## Materials and methods

### Real networks

We leverage two real-world network datasets to evaluate the generative ability of our proposed model by fitting the parameter values corresponding to each dataset. Intuitively, the aim is to find whether proposed models can mimic real-world networks efficiently. The two datasets are:

1. High Energy Physics-theory citation network (**ca- HepTh**) [49]: It is a collaboration network of scientists working in High Energy Physics-theory field. The network consists of authors as nodes and co-authorship relation between authors as an edge. It contains 8, 638 nodes and 24, 806 edges.
2. Power-grid Network (**PGN**) [14]: It is an electricity transport network in which a node represents either a generator, a transformer or a substation and an edge corresponds to a power supply line between two nodes. It contains 4, 941 nodes and 6, 594 edges.

### Multiple selection procedure

We propose a network growth mechanical model based on the idea of multiple selection procedures (with *N* = 2). In particular, the model focuses on two plausible selection procedures closely resembling the observed processes in real-world networks. We combine these real-world processes in an MSP framework leading to a more realistic and generalized evolution process of networks. An incoming node in the system connects to the existing nodes in the network based on any of the following two selection procedures:

1. Preferential attachment with aging.
2. Random attachment with local growth.

#### Preferential attachment with aging

The first procedure combines degree-based preferential attachment with self aging. At each time-step (*t*), a new node enters the system. An already existing node (*i*) with degree *k*_*i*_(*t*) attracts the new node *j* due to its preferential ability (*π*) as

$$\begin{array}{c} \pi_{i}\left(t\right)=\frac{k_{i}\left(t\right)}{t} \end{array}$$ (2)

In contrast to the above preferential mechanism, the self-aging mechanism restricts the ability of an existing node to attract incoming nodes. For example, in a paper citation network, older papers receive fewer citations than similar newer papers due to field obsolescence [50, 51]. A young node possesses more ability to attract incoming nodes than an older node [52]. Similar growth behavior was observed by Dorogovtsev et al. [24]. They found that the aging is proportional *τ*^−*α*^, where *τ* is the age of the node and *α* is the aging exponent. They also claimed that the network shows scaling behavior only in the region *α* > 1. Even though aging function can exist in several possible mathematical forms, we propose a novel variant parameterized by the current degree of the node. The intuition lies in the hypothesis that the willingness of a node to accept new connections decreases as the current number of neighbors increases. The self-aging function *f*_*i*_(*t*) is a non-increasing function of time (*t*) defined below

$$\begin{array}{c} f_{i}\left(t\right)=\frac{1}{1+b_{i}k_{i}\left(t\right)}, \end{array}$$ (3)

where, *b*_*i*_ is a positive constant that controls the rate of the aging of node *i*. As evident from Eq (3), *f*_*i*_(*t* + 1) ≤ *f*_*i*_(*t*) ∀*t* and *f*_*i*_(*t*) < 1, ∀*i* and *t* ∈ [1 ∞). The self-aging restricts the growth in the number of connections of a node as time advances. More specifically, self-aging restricts the growth of hubs in a network. Next, we combine preferential attachment (described in Eq (2)) with self-aging expression (described in Eq (3)) in single formulation *F*_*i*_(*t*) given by

$$\begin{array}{c} F_{i}\left(t\right)=\pi_{i}\left(t\right)*f_{i}\left(t\right)=\frac{k_{i}\left(t\right)}{(1+b_{i}k_{i}\left(t\right))t}=\frac{k_{i}}{(1+bk_{i})t}. \end{array}$$ (4)

The rightmost derivation in Eq (4) simplifies the nomenclature by replacing *k*_*i*_(*t*) with *k*_*i*_ and *b*_*i*_ with *b* (assuming that all nodes have similar aging rate). Next, we describe the second procedure.

#### Random attachment with local growth

The second procedure accounts random attachment with local growth. It facilitates attachment in two scenarios; direct (DRA) and indirect random attachment (IDRA). In DRA, the incoming node (*j*) attaches to an already existing node (*i*), randomly. Thus each existing node at time-step (*t*) attracts the incoming node with equal probability ($=\frac{1}{t}$). In IDRA, an initial attachment to a neighbor of node *i* favors link formation with *i* (similar to the link-copying mechanism proposed by Kumar et al. [53]). However, due to limited link formation capacity (of node *i*), the IDRA probability decreases as the degree (of node *i*) increases. We also term IDRA as “random attachment mechanism with local growth”. The above two scenarios are combined in a single formulation *ϕ*_*i*_(*t*) given by

$$\begin{array}{c} \phi_{i}\left(t\right)=\text{DRA}+\text{IDRA}, \end{array}$$ (5)

$$\begin{array}{c} \phi_{i}\left(t\right)=\frac{1}{t}+\frac{g_{i}\left(t\right)}{t}(1-\frac{1}{t}), \end{array}$$ (6)

For larger value of *t*, $(1-\frac{1}{t})\to 1$

$$\begin{array}{c} \phi_{i}\left(t\right)=\frac{1}{t}+\frac{g_{i}\left(t\right)}{t}, \end{array}$$ (7)

where 1/*t* denotes DRA probability, *g*_*i*_(*t*) is a non-negative real-valued function which associates cost of linking with the local growth. Even though local growth dynamics helps in increasing the concentration of triangles in the network, the cost of link formation restricts the growth of the degree of nodes.

#### A plausible cost function

**Fig 2:**
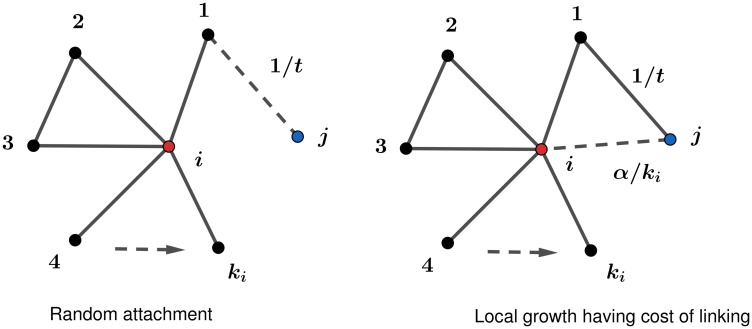
Random attachment with local growth having cost of linking. Sub-figure (a) is showing the random attachment of node *j* to a neighbour of node *i* by probability 1/*t* (first step). In sub-figure (b), after connecting to a neighbour of node *i* in the first step, node *j* has linking cost inversely proportional to the degree of node *i* under local growth.

Consider a node *i* placed under IDRA process. Node *i* has *k*_*i*_ neighbours (degree). Node *j* joins the network formation process at time (*t* + 1). Node *j* connects to a neighbour of node *i* with probability 1/*t*. Later, *j* attempts to connect with *i*. The probability of a connection between node *i* and *j* depends on the linking cost. We argue that this indirect linking cost is inversely proportional to the current degree $\left(\propto \frac{1}{k_{i}\left(t\right)}\right)$. The intuition lies in the capacity constraints in link formation [54]. In general, researchers have shown that the formation of a link can inhibit the formation of another one, typically due to time, space, or capacity constraints [55]. The process is explained in Fig 2. Node *i* has *k*_*i*_ chances to receives new links due to the local growth process of its neighbors. This results in

$$\begin{array}{c} g_{i}\left(t\right)=\alpha \sum_{x=1}^{k_{i}}\frac{1}{k_{i}}=\alpha \end{array}$$ (8)

where *α* (>0) is a proportionality constant for a network. Different networks might exhibit different values of *α*.

Consider a new node *j* randomly connects with an older node *r*, then attempts to form a link with first neighbors of node *r*. Let node *i* is one of the first neighbors of node *r* then cost of linking of nodes *i* and *j* depends on 1/*k*_*i*_ which tells that higher degree node has higher cost or low probability of being connected to a new node under local growth scheme. At the same time in the same process of link formation, for an older node *i*, 1/*k*_*i*_ is fixed for all the possibilities when a new node joins one of its neighbors and tries to connect with node *i* with probability 1/*k*_*i*_ and collectively results in a constant value *α*. In another way, we can understand the cost of linking to an older node. Constant *α* tells that an older node of higher degree has higher chances to be connected with a new joining node as compared to an older node of low degree but in both the cases gain is same which signifies the high linking cost of higher degree node.

#### MSP-2: The proposed network growth model

We combine the above two selection procedures in an MSP framework (hereafter *MSP-2*). The combined effect is reformed in terms of a differential equation using standard mean-field approximation [56] given by

$$\begin{array}{c} \frac{\partial k_{i}}{\partial t}=\beta_{j}F_{i}\left(t\right)+(1-\beta_{j})\phi_{i}\left(t\right). \end{array}$$ (9)

where *β*_*j*_ ∈ (0, 1). Here *F*_*i*_(*t*) and *ϕ*_*i*_(*t*) corresponds to expected gain in degree of node *i* due to preferential attachment with aging and random attachment with local growth, respectively. To keep the notation simple, we assume that all incoming nodes have same *β*, thus *β*_*j*_ = *β* ∀*j*.

#### The generative algorithm

1. Consider an initial connected network of *m*_0_ nodes.
2. A new node *j* joins the network.
3. Toss coin with head probability *β*_*j*_ = *β*,
  - If *head*: node *j* chooses preferential attachment mechanism with self-aging. The probability of connecting a node *i* in the pre-existing network is given by $\frac{k_{i}}{(1+bk_{i})t}.$
  - If *tail*: node *j* randomly selects a node *i* and gets connected to it. After that node *j* connects with the first neighbors of the node *i* according to the probability *α*/*k*_*l*_ ≤ 1, where *l* is a neighboring node of the node *i*.
4. Repeat steps 2and3 until the network has desired number of nodes.

## Results

Next, we analyze the degree distribution, average connectivity, and edge densification. We also explore various structural and spectral measures such as triangle count, modularity structure and spectral radius, by theoretical calculations, and numerical validations.

### The degree distribution

We, first of all, derive the generic degree distribution by combining Eqs (4), (7), (8) and (9), that results in the following formulation

$$\begin{array}{c} \frac{\partial k_{i}}{\partial t}=\beta \frac{1}{1+bk_{i}}\frac{k_{i}}{t}+(1-\beta )\frac{1+\alpha }{t}. \end{array}$$ (10)

rearranging above equation results in

$$\begin{array}{c} (\frac{k_{i}+1/b}{c_{1}(k_{i}+c_{2})})\frac{\partial k_{i}}{\partial t}=\frac{1}{t}, \end{array}$$ (11)

where *c*_1_ = *β*/*b* + (1 − *β*)(1 + *α*) and $c_{2}=\frac{\left(1-\beta )(1+\alpha \right)}{\beta +b(1-\beta )(1+\alpha )}$.

$$\begin{array}{c} \frac{1}{c_{1}}(\frac{k_{i}+c_{2}+1/b-c_{2}}{k_{i}+c_{2}})\frac{\partial k_{i}}{\partial t}=\frac{1}{t}, \end{array}$$ (12)

$$\frac{1}{c_{1}}(1+\frac{1/b-c_{2}}{k_{i}+c_{2}})\frac{\partial k_{i}}{\partial t}=\frac{1}{t},$$

$$\frac{1}{c_{1}}(1+\frac{\gamma }{k_{i}+c_{2}})\frac{\partial k_{i}}{\partial t}=\frac{1}{t},$$

where

$$\gamma =1/b-c_{2}$$

After solving the PDE given above

$$\begin{array}{c} \frac{1}{c_{1}}(k_{i}-k_{i}^{0}+\gamma \ln\frac{k_{i}+c_{2}}{k_{i}^{0}+c_{2}})=\ln\frac{t}{t_{i}} \end{array}$$ (13)

where $k_{i}^{0}$ is the initial degree of node *i* which appears at time *t*_*i*_.

$$\begin{array}{c} k_{i}-k_{i}^{0}+\gamma \ln\frac{k_{i}+c_{2}}{k_{i}^{0}+c_{2}}=c_{1}\ln\frac{t}{t_{i}} \end{array}$$ (14)

$$\ln e^{k_{i}-k_{i}^{0}}+\gamma \ln\frac{k_{i}+c_{2}}{k_{i}^{0}+c_{2}}=c_{1}\ln\frac{t}{t_{i}}$$

$$\ln\frac{e^{k_{i}}}{e^{k_{i}^{0}}}+\ln{\left(\frac{k_{i}+c_{2}}{k_{i}^{0}+c_{2}}\right)}^{\gamma }=\ln{\left(\frac{t}{t_{i}}\right)}^{c_{1}}$$

$$\ln\frac{e^{k_{i}}{\left(k_{i}+c_{2}\right)}^{\gamma }}{e^{k_{i}^{0}}{\left(k_{i}^{0}+c_{2}\right)}^{\gamma }}=\ln{\left(\frac{t}{t_{i}}\right)}^{c_{1}}$$

$$e^{k_{i}}{\left(k_{i}+c_{2}\right)}^{\gamma }\frac{1}{e^{k_{i}^{0}}{\left(k_{i}^{0}+c_{2}\right)}^{\gamma }}={\left(\frac{t}{t_{i}}\right)}^{c_{1}}.$$

By the law of large numbers, at large *t*, $e^{k_{i}^{0}}{\left(k_{i}^{0}+c_{2}\right)}^{\gamma }\to \langle e^{k_{i}^{0}}{\left(k_{i}^{0}+c_{2}\right)}^{\gamma }\rangle =\eta^{c_{1}}$ (average of expected initial degree of nodes), where *η* is a positive constant.

$$e^{k_{i}}{\left(k_{i}+c_{2}\right)}^{\gamma }\frac{1}{\eta^{c_{1}}}={\left(\frac{t}{t_{i}}\right)}^{c_{1}}.$$

Consider,

$$k<k_{i},$$

$$e^{k}{\left(k+c_{2}\right)}^{\gamma }\frac{1}{\eta^{c_{1}}}<e^{k_{i}}{\left(k_{i}+c_{2}\right)}^{\gamma }\frac{1}{\eta^{c_{1}}},$$

$$e^{k}{\left(k+c_{2}\right)}^{\gamma }\frac{1}{\eta^{c_{1}}}<{\left(\frac{t}{t_{i}}\right)}^{c_{1}},$$

$$t_{i}<\eta e^{-k/c_{1}}{\left(k+c_{2}\right)}^{-\gamma /c_{1}}t.$$

As the network is growing uniformally, the probability of selecting a node *t*_*i*_ at time t, *P*(*t*_*i*_) = 1/(*t* + *m*_0_), where *m*_0_ is the size (number of nodes) of initial seed network [56], and

$$\begin{array}{cc} P(k_{i}>k) & =P(t_{i}<\eta e^{-k/c_{1}}{\left(k+c_{2}\right)}^{-\gamma /c_{1}}t)\\ & =\frac{t}{m_{0}+t}\eta e^{-k/c_{1}}{\left(k+c_{2}\right)}^{-\gamma /c_{1}} \end{array}$$

As *t* → ∞,

$$\begin{array}{c} P\left(k_{i}>k\right)=\eta e^{-k/c_{1}}{\left(k+c_{2}\right)}^{-\gamma /c_{1}}, \end{array}$$ (15)

**Fig 3:**
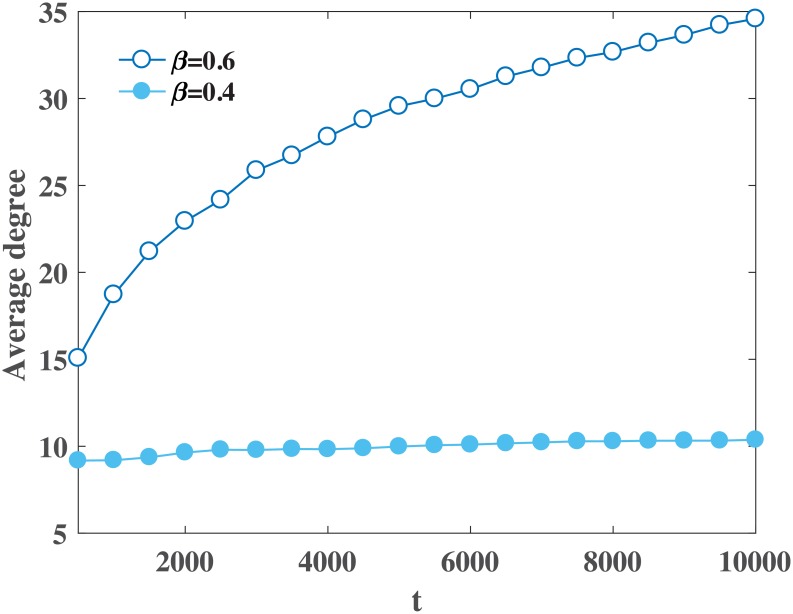
Average degree, $\bar{k}\left(t\right)$, is plotted for different size of networks simulated under MSP-2 by setting *β* = 0.4 (plot in dots) and *β* = 0.6 (plot in circles), and *α* = 1 in both the cases. For *β* = 0.4 average degree reaches to steady state (constant) and for *β* = 0.6 (> 0.5) average degree increases continuously.

The above equation represents a class of networks that demonstrate power-law degree distribution with exponential cut-off. Specifically, the two classes can be derived as follows:

- **Exponential degree distribution**: Assume that if *β* → 0, then $c_{2}\to \frac{1}{b}$ and *γ* = 1/*b* − *c*_2_ → 0. The cumulative degree distribution expression Eq (15) reduces to
  $$\begin{array}{c} P\left(k_{i}>k\right)\to \eta e^{-k/(1+\alpha )}, \end{array}$$ (16)
  Eq (16) refers to the networks of exponential degree distribution with $k_{j}^{0}\to \left(1+\alpha \right)$ and average degree $\bar{k}\to 2\left(1+\alpha \right)$.
- **Scale-free degree distribution**: Assume that if *b* = 0, then from Eq (10)
  $$\begin{array}{c} \frac{\partial k_{i}}{\partial t}=\beta (\frac{k_{i}}{t})+(1-\beta )(\frac{1+\alpha }{t}), \end{array}$$ (17)
  and
  $$\begin{array}{c} P\left(k_{i}>k\right)\propto {\left(k+(1-\beta )(1+\alpha )/\beta \right)}^{-1/\beta }. \end{array}$$ (18)
  Eq (18) refers to the class of scale-free networks that follow power-law degree distribution with $k_{j}^{0}=\beta {\bar{k}}_{t}+\left(1-\beta \right)\left(1+\alpha \right)$ and
  $${\bar{k}}_{t+1}=\frac{t{\bar{k}}_{t}+2k_{j}^{0}}{t+1}=\frac{t{\bar{k}}_{t}+2\beta {\bar{k}}_{t}+2\left(1-\beta \right)\left(1+\alpha \right)}{t+1},$$
  $$\begin{array}{c} {\bar{k}}_{t+1}={\left(t+1\right)}^{2\beta -1}(1+\theta )-\theta , \end{array}$$ (19)
  where ${\bar{k}}_{t}$ is the average connectivity of the network at time *t*, $\theta =\frac{2(1-\beta )(1+\alpha )}{\left(2\beta -1\right)}$ and *β* ∈ (0, 1). This represents a densification power law (DPL) [57], exhibiting phase transition at *β* = 1/2. For *β* > 1/2, the average degree increases as the network evolves over the period of time and for *β* ≤ 1/2 average degree of evolving network approaches the limiting value of 2(1 − *β*)(1 + *α*)/(1 − 2*β*) as *t* → ∞ (see Fig 3).

### The average connectivity

Next, we investigate the dynamics of the average connectivity of MSP-2. At each time step, a node arrives and attaches itself to pre-existing nodes in the network. Consider node *j* being introduced in the network formation process at the time step (*t* + 1). From Eq (10), the initial degree of node *j* is given by

$$\begin{array}{c} k_{j}^{0}=\sum_{i\in N_{t}}(\beta (\frac{1}{1+bk_{i}}\frac{k_{i}}{t})+(1-\beta )(\frac{1+\alpha }{t})). \end{array}$$ (20)

Average connectivity of the network at time *t* + 1 will be

$$\begin{array}{cc} {\bar{k}}_{t+1}= & \frac{t{\bar{k}}_{t}+2k_{j}^{0}}{t+1},\\ {\bar{k}}_{t+1}= & \frac{t{\bar{k}}_{t}+2\left(1-\beta \right)\left(1+\alpha \right)+2\beta /b-\frac{2\beta }{b}\sum_{i}\frac{1}{(bk_{i}+1)t}}{t+1},\\ {\bar{k}}_{t+1}\geq & \frac{t{\bar{k}}_{t}+2\left(1-\beta \right)\left(1+\alpha \right)+2\beta /b-\frac{2\beta }{b}\sum_{i}\frac{1}{(1+b)t}}{t+1},\\ {\bar{k}}_{t+1}-{\bar{k}}_{t}\geq & \frac{-{\bar{k}}_{t}+2\frac{\beta }{b}+2\left(1-\beta \right)\left(1+\alpha \right)-\frac{2\beta /b}{1+b}}{t+1},\\ \frac{\partial {\bar{k}}_{t+1}}{\partial t}\geq & \frac{-{\bar{k}}_{t}+2\frac{\beta }{b}+2\left(1-\beta \right)\left(1+\alpha \right)-\frac{2\beta /b}{1+b}}{t+1}. \end{array}$$ (21)

The solution of Eq (21) is given by,

$${\bar{k}}_{t+1}\geq {\left(t+1\right)}^{-1}(1-{\bar{k}}_{L})+{\bar{k}}_{L},$$

where ${\bar{k}}_{L}=\frac{2\beta }{1+b}+2\left(1-\beta \right)\left(1+\alpha \right)$. As $t\to \infty \Rightarrow {\bar{k}}_{\infty }\to {\bar{k}}_{L}$. Similarly,

$${\bar{k}}_{t+1}\leq {\left(t+1\right)}^{-1}(1-{\bar{k}}_{U})+{\bar{k}}_{U},$$

where ${\bar{k}}_{U}=2\beta /b+2\left(1-\beta \right)\left(1+\alpha \right)$. As $t\to \infty \Rightarrow {\bar{k}}_{\infty }\to {\bar{k}}_{U}$. ${\bar{k}}_{L}$ and ${\bar{k}}_{U}$ are lower and upper bounds of the average connectivity of the network model defined by Eq (10).

### Edge densification

Edge densification prevents the growth of the diameter of networks generated under the proposed model. Consider two networks with same number of nodes corresponding to *β*_1_ and *β*_2_ (≥ *β*_1_) (discussed in Eq (19)), MSP-2 generate networks with different average connectivity and different diameter growth rate. Let *D*_1_ and *D*_2_ be the diameters of the networks generated under Eq (19) by setting *β* = *β*_1_ and *β*_2_, respectively, such that *β* ≥ 1/2 and *b* = 0. The proposed modelling scheme results in networks with *D* ∝ (1 − *β*) (proof, similar to [33], is omitted due to space constraints). Again consider

$$\begin{array}{c} \beta_{1}\leq \beta_{2}\Rightarrow \bar{k}\left(\beta_{1}\right)\leq \bar{k}\left(\beta_{2}\right),\\ \beta_{1}\leq \beta_{2}\Rightarrow 1-\beta_{1}\geq 1-\beta_{2}\Rightarrow D_{1}\geq D_{2}. \end{array}$$

So, if *β* has different values and keeping the rest of the parameters constant in Eq (19), then

$$\bar{k}\left(\beta_{1}\right)\leq \bar{k}\left(\beta_{2}\right)\Rightarrow \beta_{1}\leq \beta_{2}\Rightarrow D_{1}\geq D_{2}.$$

### Modularity or community structure

Real-world networks inherit community structure. Community is a group of nodes possessing more number of links than expected in random networks [18]. In the context of social structure, a community is a group of similar people having a significantly high number of connections within the community and lesser connections to the outside world. To measure the quality of community structure inside a network, modularity index *Q* [18] is defined as

$$Q=\frac{1}{2m}\sum_{ij}[A_{ij}-\frac{k_{i}k_{j}}{2m}]\delta_{c_{i}c_{j}}$$

where *m* is the number of edges in the network, *k*_*i*_ and *k*_*j*_ are the degrees of the nodes *i* and *j* which belong to communities *c*_*i*_ and *c*_*j*_ respectively, and *δ* is the Kronecker delta function.

Next, we conduct the theoretical analysis of the possibility of community structure in MSP-2. We argue that similar results hold for higher-order MSP variations (*N* > 2). Consider the connection probability *p*_*ij*_ between two nodes *i* and *j* which appear at time *t*_*i*_ and *t*_*j*_(> *t*_*i*_), respectively

$$p_{ij}=\beta \frac{k_{i}}{1+bk_{i}}\frac{1}{t}+\left(1-\beta \right)\frac{1+\alpha }{t}.$$

As we know that at each time a new appears, so *t* = *t*_*j*_ and we get

$$p_{ij}=\beta \frac{k_{i}}{1+bk_{i}}\frac{1}{t_{j}}+\left(1-\beta \right)\frac{1+\alpha }{t_{j}}.$$

Here, (1 + *bk*_*i*_) is applied to restrict the growth of the degree of node *i*. Hence, without loss of generality, we consider a simpler case, where *b* = 0. We claim that similar results true for other cases of the proposed model. We consider

$$\begin{array}{c} p_{ij}=\beta \frac{k_{i}\left(t_{j}\right)}{t_{j}}+\left(1-\beta \right)\frac{1+\alpha }{t_{j}}. \end{array}$$ (22)

and after solving PDE Eq (17), we have

$$\begin{array}{c} \begin{array}{cc} k_{i}\left(t_{j}\right) & =c_{i}^{0}{\left(\frac{t_{j}}{t_{i}}\right)}^{\beta }-\frac{\left(1-\beta )(1+\alpha \right)}{\beta },\\ c_{i}^{0} & =(k_{i}^{0}+\frac{\left(1-\beta )(1+\alpha \right)}{\beta }). \end{array} \end{array}$$ (23)

Let *A* be the adjacency matrix associated with a network *G*, where *A*_*ij*_ = 1, if nodes *i* and *j* are connected otherwise 0. Corresponding *null-model* is defined in the following way: if *k*_*i*_ and *k*_*j*_ are the degrees of nodes *i* and *j*, respectively, and *m* is the number of edges in the network *G* then *k*_*i*_*k*_*j*_/2*m* is the expected value of *A*_*ij*_ under null-model and community structure is measured by the non-zero heterogeneous values of $A_{ij}-\frac{k_{i}k_{j}}{2m}$. Here, we have only expected values of *A*_*ij*_ of a network obtained under MSP-2 that is *E*[*A*_*ij*_] = *p*_*ij*_. Using Eqs (22) and (23), we have

$$\begin{array}{c} E\left[A_{ij}\right]=\frac{\beta c_{i}^{0}}{t_{i}^{\beta }t_{j}^{1-\beta }} \end{array}$$ (24)

and

$$\begin{array}{c} \frac{k_{i}k_{j}}{2m}=\{\begin{array}{cc} \frac{c_{i}^{0}c_{j}^{0}}{\left(1+\theta \right)t_{j}^{\beta }t_{i}^{\beta }} & \text{if}\;\;\beta \geq 1/2\\ \frac{c_{i}^{0}c_{j}^{0}}{\theta t_{j}^{\beta }t_{i}^{\beta }t^{1-2\beta }} & \text{if}\;\;\beta <1/2 \end{array} \end{array}$$ (25)

where $c_{i}^{0}$ and $c_{j}^{0}$ are initial conditions. As we know that *β* controls the contribution of two micro-level network growth processes in the resulting evolution of the network, so we analyze the effect of the parameter *β* over the strength of community structure of the obtained network under the proposed model. We consider the condition when *β* approaches to 1 (main contribution made by preferential attachment scheme), and have

$$\begin{array}{c} p_{ij}=E\left[A_{ij}\right]=\frac{\beta c_{i}^{0}}{t_{i}^{\beta }t_{j}^{1-\beta }} \end{array}$$ (26)

and as $\beta \to 1\Rightarrow c_{j}^{0}\to k_{j}^{0}\to \beta {\bar{k}}_{t_{j}}\to t_{j}^{2\beta -1}$, *θ* → 0 that results

$$\begin{array}{c} \frac{k_{i}k_{j}}{2m}∼\frac{\beta c_{i}^{0}}{t_{i}^{\beta }t_{j}^{1-\beta }} \end{array}$$ (27)

**Fig 4:**
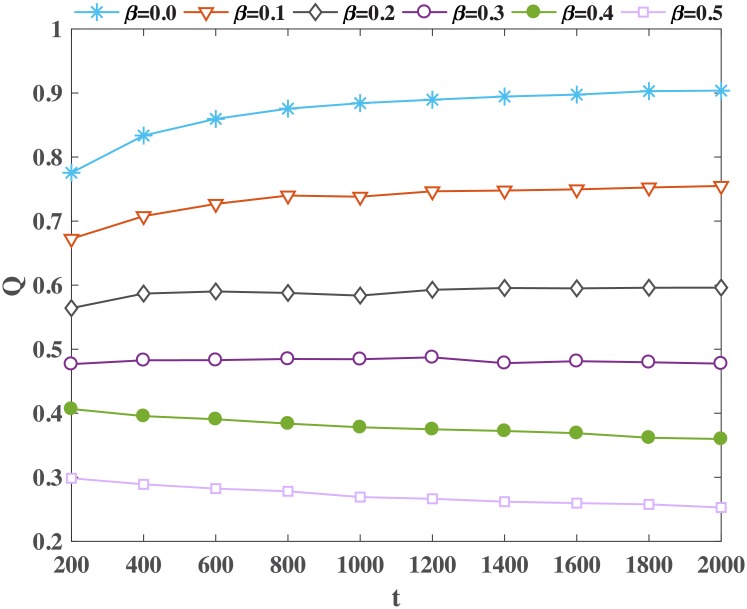
Modularity index, Q, is plotted for different size of networks generated by the proposed model with different values of *β*. Horizontal-axis represents the number of nodes in the networks and the vertical-axis represents modularity index of the networks.

From Eqs (26) and (27), when *β* approaches to 1, the distribution of links (*p*_*ij*_) in the network simulated under the proposed model approaches to null model ($\frac{k_{i}k_{j}}{2m}$) and strength of community structure (*Q*) gets reduced, see Fig 4.

Again we consider another condition when *β* approaches to 0, and have

$$\begin{array}{c} p_{ij}=E\left[A_{ij}\right]=\frac{1+\alpha }{t_{j}} \end{array}$$ (28)

and

$$\begin{array}{c} \frac{k_{i}k_{j}}{2m}∼\frac{1+\alpha }{t}\log\frac{t}{t_{i}}\log\frac{t}{t_{j}} \end{array}$$ (29)

From Eqs (28) and (29), when *β* approaches to 0, the distribution of links (*p*_*ij*_) are different from null model ($\frac{k_{i}k_{j}}{2m}$) and strong community structure appears, see Fig 4.

Modularity index *Q* is defined over matrix *B*, where

$$B_{ij}=A_{ij}-\frac{k_{i}k_{j}}{2m}∼E\left[A_{ij}\right]-\frac{k_{i}k_{j}}{2m}=p_{ij}-\frac{k_{i}k_{j}}{2m}.$$

We discuss the strength of modularity in terms of positive eigenvalues of matrix *B*. We have already shown two extreme ends when *β* = 1 and *β* = 0. As *β* approaches to 1 $B_{ij}=p_{ij}-\frac{k_{i}k_{j}}{2m}$ approaches to 0 and maximum eigenvalue approaches to 0 and weak community structure arises. In another direction, when *β* approaches to 0 we have heterogeneous values of $B_{ij}=p_{ij}-\frac{k_{i}k_{j}}{2m}$, and eigenvalues spread over a wide range (it is true due to constant volume of matrix *B*) and gets higher maximum eigenvalue of *B*, close to 1 (strong community structure). While finding communities in a network, we decompose the network into small sub-networks that is equivalent to decomposition of matrix *B* in such a way that diagonal of *B* has blocks of positive entries and off-diagonal blocks have maximum negative entries, and it can happen only when *B* has heterogeneous structure which appears when *β* has lower value. We can conclude that local dynamics is responsible for community structure.

The distribution of expected links in the proposed model is different from the corresponding distribution of *k*_*i*_*k*_*j*_/2*m* values. If the probability of the existence of a link between nodes *i* and *j* is different from the value *k*_*i*_*k*_*j*_/2*m*, which is the probability of the existence on a link between nodes *i* and *j* under null model, then the structure of the network obtained under the model would be different as compared to the expected null structure (under null model) for given degree sequence. This leads to the existence of community structure in the network (higher value of maximum eigenvalue of *B* explained in the previous paragraph). As the *β* reduces, the heterogeneity of $E\left[A_{ij}\right]-\frac{k_{i}k_{j}}{2m}$ increases and network generated under MSP-2 shows strong community structure. Fig 4 demonstrates the modularity index of the networks generated by the proposed model. It clearly shows the existence of the community structure. The contribution of local dynamics, (1 − *β*), in the network evolution, affects the community structure of the resulting network positively. A smaller value of *β* does not indicate that the network is denser as we have the condition that if (2*β* − 1) is greater than 1/2 then network would show densification. However, as *β* decreases (1 − *β*) increases but (2*β* − 1) decreases. Theoretical analysis and numerical simulation show similar results. As *β* decreases, the value of modularity index (Q) increases (see Fig 4). It is needless to mention that this phenomenon is common in various social networks [58]. We leverage Louvain algorithm [59] to detect community structures in networks.

### Number of triangles

In a network, a triangle is a cycle of three nodes. The high concentration of triangles in a network is a fundamental property of many real networks. In real-world networks, social phenomena such as *“friends of a friend are friends”* beautifully explain the high concentration of triangles. Several social networks have a high density of triangles, for example, ego-Facebook network, ego-Gplus network, and ego-Twitter [7].

We provide a lower bound to estimate the number of triangles for the proposed model. As evident from previous discussions, the local dynamics of network evolution is responsible for the triangle generation. Consider an existing node *i* with degree *k*_*i*_ and an incoming node *j*. In MSP-2, triangle generation occurs in the following two scenarios:

- Node *j*, first, connects to one of neighbours of node *i* with probability 1/*t*. Later, *j* connects to *i* with probability *α*/*k*_*i*_. Thus, node *i* has *k*_*i*_ chances to form a triangle, each with probability $\frac{\alpha }{k_{i}t}$.
- Similarly, node *j* can first link to node *i* with probability 1/*t* and then connects with the neighbours of *i* with probability *α*/*k*_*l*_, where node *l* is the neighbour of node *i* other than *j*.

Next, we compute expected number of triangles generated by above two scenarios at time *t*. At each time step, network generates atleast *Tr* triangles, given by

$$\begin{array}{c} Tr\left(t\right)=\sum_{i=1}^{t}\frac{(1-\beta )\alpha }{t}+\sum_{i=1}^{t}\frac{(1-\beta )\alpha }{t}\sum_{l=1}^{t}A_{il}\frac{1}{k_{l}}=2\left(1-\beta \right)\alpha . \end{array}$$

The expected number of total triangles in the network is given by

$$\Delta_{t}\geq 2\left(1-\beta \right)\alpha t.$$

The number of triangles in a network produced by the proposed model is lower bounded by a linear function which has slope 2(1 − *β*)*α*, and *β* controls the density of triangles in the model generated networks.

**Fig 5:**
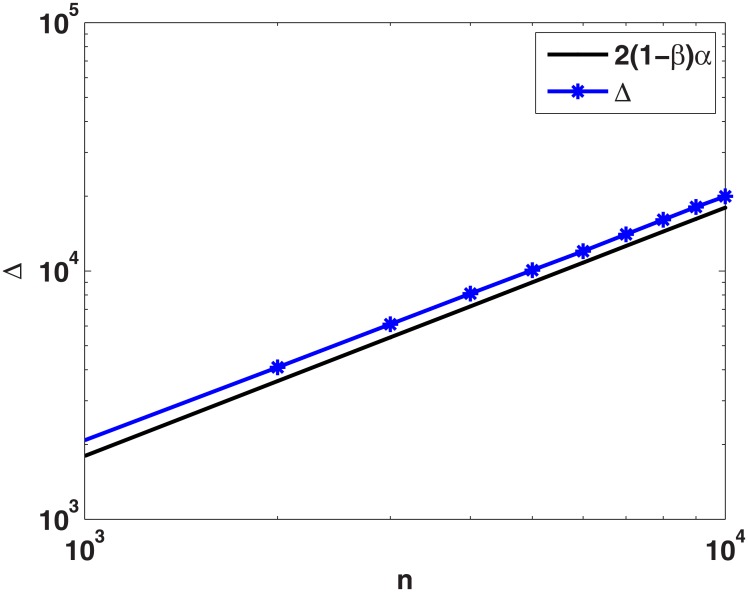
Number of triangles (Δ) is plotted for different size of networks generated by the proposed model by setting *β* = 0.1, *b* = 1, and *α* = 1.0. Theoretically calculated lower bound (2(1 − *β*)*α*) is also plotted. Horizontal-axis represents the number of nodes in the networks and the vertical-axis represents number of triangles in the networks.

Simulation experiments validate the above theoretical bounds. We generate networks using MSP-2 by setting *β* = 0.1, *α* = 1 and *b* = 1. The theoretical and numerical results are plotted in Fig 5. The theoretical result provides a good estimate of numerical values for triangle count in the networks generated under MSP-2.

### Spectral radius

The largest eigenvalue of an adjacency matrix associated with a network is known as the spectral radius (SR) of the network. Empirically, the reciprocal of SR quantifies the threshold of viral propagation in the network [60]. The networks with smaller spectral radius have larger robustness against the spread of viruses [60]. We derive bounds on SR for the networks produced under MSP-2. The bounds on SR can be leveraged to attain bounds on diffusion threshold. Let λ_1_(*A*) is the largest eigenvalue of the adjacency matrix *A* associated with a network produced by MSP-2. From [61], we know that

$$\begin{array}{c} k_{\text{max}}\geq \lambda_{1}\left(A\right)\geq \bar{k}, \end{array}$$ (30)

where *k*_max_ is the maximum degree and $\bar{k}$ is the average degree of the network.

By Eq (13), we get the expected value of maximum degree in a network of size *n* nodes obtained under MSP-2 by setting $k_{i}^{0}=1$, *t*_*i*_ = 1 and *t* = *n* that is

$$k_{\text{max}}+\gamma \ln\left(k_{\text{max}}+c_{2}\right)=1+\gamma \ln\left(1+c_{2}\right)+c_{1}\ln n.$$

After simplifications and approximations, we get

$$\begin{array}{c} 1+c_{1}\ln n\geq k_{\text{max}}\geq \frac{c_{1}}{1+\gamma }\ln n. \end{array}$$ (31)

By Eqs (30) and (31) and lower bound on average connectivity ${\bar{k}}_{L}$ (Eq (21))

$$1+c_{1}\ln n\geq \lambda_{1}\left(A\right)\geq \frac{2\beta }{b+1}+2\left(1-\beta \right)\left(1+\alpha \right).$$

SR of a growing network under MSP-2 has a growth rate lower than the logarithm of the size of the network. Using the bounds on SR, we can select the model parameters to generate a network of the desired property.

### Reconstructing real networks

As discussed earlier, the majority of the real-world networks follow a combination of multiple degree distributions and not just power-law distribution [5, 6]. The neuronal network of the worm *C. elegans* and the power-grid network of Southern California shows exponential decay in cumulative connectivity of the nodes [5] while networks of scientific collaborators exhibit power-law with exponential cut-off [6].

#### Parameter tuning

Assume that we produce a model network corresponding to a real network which has Δ_*r*_ triangles, *k*_max_ maximum degree and *n* number of nodes. We know that, if *β* and *α* are model parameters then the network obtained under the proposed model would have at-least *α*(1 − *β*)*n* triangles. We consider the relation

$$\begin{array}{c} \frac{\Delta_{r}}{n}=\alpha \left(1-\beta \right), \end{array}$$ (32)

to ensure that the reconstructed network has at-least Δ_*r*_ triangles. We have another relation between maximum degree and the size of the network.

$$\begin{array}{c} e^{k_{\text{max}}}{\left(k_{\text{max}}+c_{2}\right)}^{\gamma }\frac{1}{e{\left(1+c_{2}\right)}^{\gamma }}=n^{c_{1}} \end{array}$$ (33)

First we discretize the interval [0, 1], and let say *S* be the set of those discrete points. Select *β* ∈ *S* and using Eq (32) compute the value of *α*. Again, using the obtained values of *α* and *β*, we compute the value of *b* by solving Eq (33), numerically. Now, we simulate model for the computed values of (*β*, *α*, *b*) and select the model network which has minimum value of |*P*(*k*_*i*_ ≥ *k*) − *P*_*r*_(*k*_*i*_ ≥ *k*)| for the set *S*, where *P*(*k*_*i*_ ≥ *k*) and *P*_*r*_(*k*_*i*_ ≥ *k*) are cumulative degree distributions of model network and given real network, respectively.

**Fig 6:**
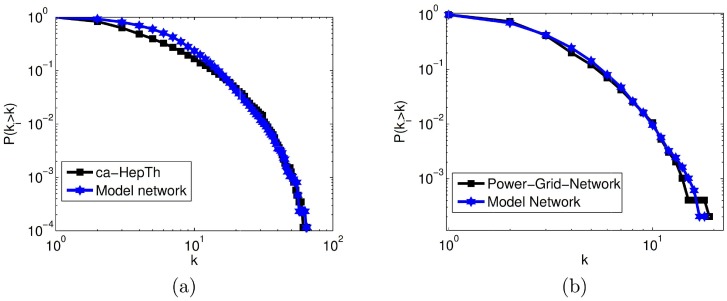
(a) Cumulative degree distribution of collaboration network (in black) and Model network (in blue). The parameter values are *β* = 0.15, *α* = 3.8 and *b* = 0.05. (b) Cumulative degree distribution of power grid network (in black) and Model network (in blue). The parameter values are *β* = 0.5, *α* = 0.26 and *b* = 0.1.

Here, we reconstruct two real networks described in Material and Methods section; one is a collaboration network (*ca* − *HepTh*), which is an example of power-law degree distribution with exponential cut-off [49] and second is a Power-Grid-Network (PGN) which has exponential degree distribution. Cumulative degree distribution of both the networks (in black squares) and corresponding model networks (in blue stars) are plotted in Fig 6a and 6b, respectively. The model parameters are tuned as *β* = 0.15, *α* = 3.8 and *b* = 0.05 to generate a network corresponding to ‘*ca* − *HepTh*’ network. Similarly, *β* = 0.5, *α* = 0.26 and *b* = 0.1 are the parameter values to generate model network corresponding to PGN network. The overlapping of the plots in Fig 6a and 6b clearly shows that the MSP-2 generative mechanism is significantly capable enough to capture the degree distribution of different classes of real-world networks. In Fig 6a and 6b, the degree distribution of model networks are corresponding to a single snapshot. Apart from degree distribution, we compare other statistical properties such as triangles’ count Δ, spectral radius *SR*, clustering coefficient *CC*, and modularity index *Q* of real networks and corresponding model networks. Results are tabulated in Table 1. It is observed that the proposed model produces networks which have statistical properties close to real data, for example, low-spectral radius, large triangles’ count, clustering, and modularity index. It is more generalized as compared to the other previous growth models.

**Table 1:** Structural and spectral properties of real-world networks and corresponding synthetic networks obtained under our proposed model. Values in brackets represent proposed model properties.

	ca-HepTh (MSP-2)	PGN (MSP-2)
*SR*	31 (20.3)	7.5 (5.9)
Δ	27869 (38251)	651 (646)
*CC*	0.48 (0.39)	0.08 (0.11)
*Q*	0.72 (0.66)	0.93 (0.75)

## Discussion

The previous section demonstrates how well the MSP-2 mechanism can imitate real-world structural properties. Also, the proposed model is useful in generating several types of networks under different conditions depending on the settings of the parameters in Eq (10). Intuitively, we can generate the following three classes of networks conditioned on the selection procedure and parameter initialization.

**Fig 7:**
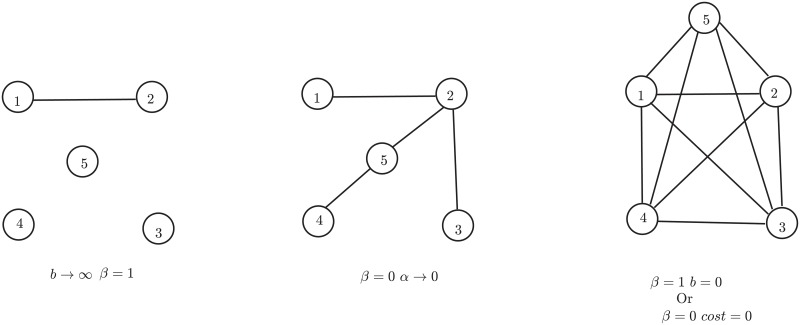
*β* = 1 and *b* → ∞ (left most network). *β* = 0 and *g*(*t*) → 0, very high cost of link formation (network in the middle). *β* = 1 and *b* = 0, or *β* = 0 and cost of link formation is zero (right most network).

- ***Empty network***: If all incoming nodes consider only the first branch of the MSP-2 (*β* = 1) along with high aging factor (*b* → ∞), then an empty network with a single edge will be generated. The generated network is closer to an extreme of the network structures (see Fig 7a).
- ***Tree network***: If all incoming nodes consider only the second branch of the MSP-2 (*β* = 0) and the cost of linking under local growth is very high, then a tree network is generated (see Fig 7b).
- ***Complete network***: Again, consider that the incoming nodes consider only the second branch of the MSP-2 (*β* = 0). If the cost of the local connection is zero, then it will generate a complete network (see Fig 7c). It is another extreme of the network topology of the possible network structures.
  If all incoming nodes only consider preferential attachment without aging (*β* = 1 and *b* = 0), then the generated network would be almost complete. However, node aging affects its growth during network evolution. *b* ≠ 0 bounds the degree of the node and average degree of the network.

All the network structures lie between the two extremes, the empty network, and the complete network. Most of the real-world networks lie between the tree and complete network topology. The simulated results described in the previous section show the potential of MSP-2 to reconstruct real-networks from different classes structurally.

## Conclusion

In this work, we propose a novel random network growth model (MSP-2) based on the observed phenomena of link formation. MSP-2 combines concepts of preferential attachment, random selection, aging, and the cost of link formation in a single process of network evolution. The combined dynamics leads to non-trivial properties exhibited by real-world networks. The proposed model successfully generates the networks corresponding to the real-world networks, like collaboration network and Power-Grid-Network. We find that the degree distribution of the real-world networks is significantly closer to the corresponding modeled networks. The properties of the networks generated under the proposed model are similar to the real-world networks. We also show that densification in a random network restricts the growth of diameter of that network. In the future, more generalized cost, and aging functions can be implemented using complex MSP-N (N>2) to incorporate various other physical phenomena.
